# Low prevalence of antibodies against *Toxoplasma gondii* in dairy cattle from China’s central region

**DOI:** 10.1186/s12917-018-1629-3

**Published:** 2018-10-19

**Authors:** Hui Dong, Yao Yao Lu, Rui Jing Su, Ying Hua Wang, Meng Yao Wang, Yi Bao Jiang, Yu Rong Yang

**Affiliations:** 1 College of Animal Science and Veterinary Medicine, Henan Agricultural University, Zhengzhou, People’s Republic of China; 2 Center for Animal Disease Control and Prevention of Henan Province, Zhengzhou, China

**Keywords:** *Toxoplasma gondii*, Cattle, Seroprevalence, Modified agglutination test, Risk factor, Henan Province, China

## Abstract

**Background:**

*Toxoplasma gondii* is an intracellular protozoan that can infect humans and other animals, including cattle. Cattle are one of the world’s main sources of meat, and people who consume raw or undercooked meat and milk of cattle infected with *T. gondii* can become infected. In this study, a total of 5292 dairy cattle serum samples, collected from 17 cities (Henan Province, China) from January 2015 to September 2017, were screened for antibodies against *T. gondii*.

**Results:**

Antibodies to *T. gondii* were found in 1.93% (102/5292) (95% CI, 1.56–2.30) of dairy cattle using a modified agglutination test (cut-off 1:100). The results showed that geographic location and season may be risk factors for *T. gondii* infection of cattle (*P <* 0.05), and the seroprevalence of *T. gondii* in cattle along the Yellow River is higher than other areas.

**Conclusions:**

This is the first large-scale investigation on the seroprevalence of *T. gondii* infection in cattle from Central China. This survey shows that the *T. gondii* infection rate of dairy cattle is low; however, these findings provide additional information on the epidemiology of Chinese *T. gondii*. The possibility of dairy cattle exposure to *T. gondii* in Central China can not be ignored, and the consumption of raw or undercooked beef or milk may pose a risk to human health.

## Background

*Toxoplasma gondii* is an intracellular protozoan parasite that can infect a wide variety of host species, including cattle [1]. The seroprevalence of *T. gondii* infection varies among host species. Although cattle appear to be poor hosts for *T. gondii*, *T. gondii* can still infect cattle and viable *T. gondii* strain had been isolated from the intestines of naturally infected cows [2, 3]. Cattle infected with *T. gondii* pose a risk for toxoplasmosis in people who consume raw or undercooked meat and unpasteurized milk [4]. In humans, *T. gondii* can cause encephalitis, retinitis, newborn hydrocephalus [1, 5], and even death [6].

Approximately 211 million cattle are raised in China, accounting for 14.4% of the world’s cattle population (National Bureau of Statistics of China, 2015 update). Surveys regarding *T. gondii* infection in cattle have been reported in some parts of China. However, only three reports on cattle *T. gondii* infections in the central region of China have been published to date, which indicated that the prevalence of *T. gondii* was 20.10% (79/393) in 2011–2013 by IHA (95% CI 16.14–24.06) [7], 5.38% (43/800) in 2011–2012 by IgG test paper (95% CI 3.81–6.94) [8], and 0 (0/102) in 2013–2014 by IHA [9], and there requires more reports as a support to summarize and analyze the epidemiological situation in the region. Large quantities of milk and meat are consumed each year in China, and the safety of the cattle products with respect to *T. gondii* infection is unknown. The objective of this investigation was to estimate the seroprevalence and risk of *T. gondii* infection in dairy cattle from Central China. To our knowledge, the present study is the most extensive investigation of *T. gondii* infections in dairy cattle from Central China.

## Methods

### Investigation site and serum samples

**Fig. 1:**
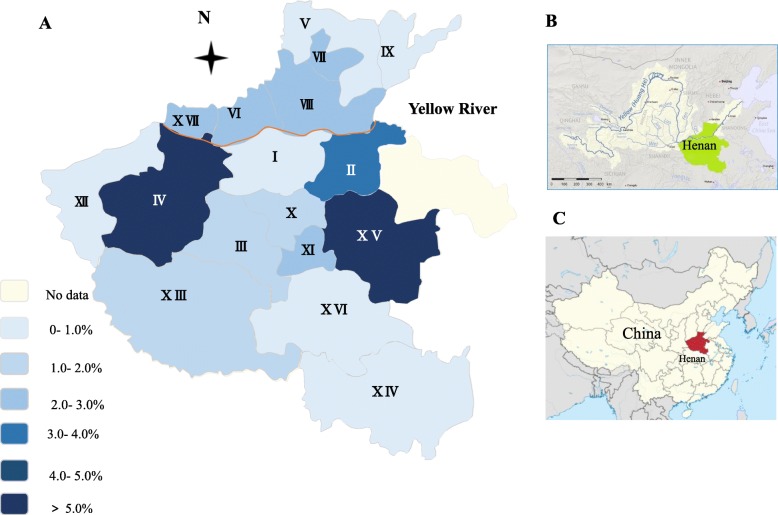
Location of samples received from Henan Province of China. Figures were adapted from Wikipedia. **a** Cities in Henan Province I: ZhengZhou, II: KaiFeng, III: PingDingShan, IV: LuoYang, V: AnYang, VI: JiaoZuo, VII: HeBi, VIII: XinXiang, IX: PuYang, X: XuChang, XI: LuoHe, XII: SanMenXia, XIII: NanYang, XIV: XinYang, XV: ZhouKou, XVI: ZhuMaDian, XVII: JiYuan. Yellow line is the Yellow River. **b** The Yellow River area in China (adapted from Wikipedia). **c** Henan Province in China (adapted from Wikipedia)

Henan Province is located in the central region of China. Henan Province (latitude 34.90°N, longitude 113.50°E) has a humid and subtropical climate. From east to west, the plains transition into the hilly mountains. The average annual temperature is 15.7 °C to 12.1 °C, and the average annual precipitation is 1380.6 to 532.5 mm. The sera of 5292 dairy cattle from 49 farms in 17 cities were collected by local veterinarians from January 2015 to September 2017 (Table 1, Fig. 1). Cattle feed consists of silage, hay, and fresh grass. The cattle were females, and their ages ranged from 2 to 15 years. The farm names and sample collection dates were recorded. The sera were used for *Brucellosis* screening, which in turn also allowed us to survey for *T. gondii* infection. The cattle sera were separated from jugular vein blood and transported to the Laboratory of Veterinary Pathology, Henan Agricultural University (Zhengzhou, Henan, China) in cooler boxes. The samples were stored at 4 °C and tested for *T. gondii* antibodies within one week.

**Table 1:** Seroprevalence of *T. gondii* in cattle in Henan Province

Location/ City	Samples obtained date	Tested No.	No. of seropositive samples /(titer)						% (Positive No. /Test No.)	95% Cl
			1:100	1:200	1:400	1:800	1:1600	1:3200		
I ZhengZhou	24 Sep 2015^b^	209	2	–	–	–	–	–	0.54% (2/369)	0.02–2.09
	21 Apr 2017	30	–	–	–	–	–	–		
	01 Aug 2017	130	–	–	–	–	–	–		
II KaiFeng	19 Oct 2015	42	3	–	–	–	–	–	3.49% (3/86)	0.77–10.18
	24 Sep 2015	44	–	–	–	–	–	–		
III PingDingShan	25 Sep 2015	300	7	3	2	2	1	–	1.98% (7/353)	0.88–4.12
	27 Jun 2017	4	–	–	–	–	–	–		
	07 Jun 2017	49	–	–	–	–	–	–		
IV LuoYang	22 Sep 2015	280	31	23	12	9	6	6	10.00% (31/310)	7.10–13.88
	22 Mar 2017	30	–	–	–	–	–	–		
V AnYang	15 Jan 2015	369	2	–	–	–	–	–	0.41% (2/488)	0.01–1.58
	05 Jul 2016	4	–	–	–	–	–	–		
	25 Apr 2017	32	–	–	–	–	–	–		
	07 Jun 2017	31	–	–	–	–	–	–		
	28 Jun 2017	22	–	–	–	–	–	–		
	29 Jun 2017	30	–	–	–	–	–	–		
VI JiaoZuo	16 Oct 2015	200	7	–	–	–	–	–	2.29% (7 /306)	1.02–4.74
	22 Apr 2017	29	–	–	–	–	–	–		
	26 Jun 2017	29	–	–	–	–	–	–		
	05 Sep 2017	48	–	–	–	–	–	–		
VII HeBi	15 Jan 2015	552	12	2	2	2	2	–	2.07% (12/581)	1.14–3.62
	15 Jun 2017	29	–	–	–	–	–	–		
VII XinXiang	16 Oct 2015	452	11	2	1	1	–	–	2.28% (11/482)	1.23–4.09
	22 Mar 2017	30	–	–	–	–	–	–		
IX PuYang	15 Oct 2015	250	2	–	–	–	–	–	0.71% (2/283)	0.02–2.71
	05 Sep 2017	33	–	–	–	–	–	–		
X XuChang	25 Sep 2015	200	5	2	2	2	2	–	1.77% (5/282)	0.64–4.20
	26 Apr 2017	52	–	–	–	–	–	–		
	27 Apr 2017	30	–	–	–	–	–	–		
XI LuoHe	25 Sep 2015	50	1	–	–	–	–	–	2.00% (1/50)	0.01–11.47
XII SanMenXia	23 Sep 2015	81	–	–	–	–	–	–	0 (0/150)	–
	21 Apr 2017	23	–	–	–	–	–	–		
	07 Sep 2017	46	–	–	–	–	–	–		
XIII NanYang	14 Oct 2015	170	2	–	–	–	–	–	1.06% (3/283)	0.21–3.22
	21 Mar 2017	16	–	–	–	–	–	–		
	22 Mar 2017	39	–	–	–	–	–	–		
	27 Jun 2017	29	–	–	–	–	–	–		
	07 Sep 2017	29	1	–	–	–	–	–		
XIV XinYang	18 Apr 2017	31	–	–	–	–	–	–	0 (0/31)	–
XV ZhouKou	19 Oct 2015	50	2	1	–	–	–	–	5.63% (4/71)	1.80–14.03
	21 Mar 2017	21	2	2	2	1	1	1		
XVI ZhuMaDian	25 Apr 2017	31	–	–	–	–	–	–	0.76% (1/131)	0.01–4.62
	29 Jun 2017	10	–	–	–	–	–	–		
	06 Sep 2017	90	1	–	–	–	–	–		
XVII JiYuan	24 Sep 2015	250	6	1	–	–	–	–	2.50% (7/280)	1.11–5.17
	22 Mar 2017	30	1	1	–	–	–	–		
XVII NK^a^	14 Oct 2015	657	4	3	–	–	–	–	0.53% (4/756)	0.15–1.40
	16 Oct 2015	50	–	–	–	–	–	–		
	04 Jul 2017	49	–	–	–	–	–	–		
Total		5292	102	40	21	17	12	7	1.93% (102/5292)	1.59–2.34

^a^NK means the city information of samples were not known

^b^The samples sampled on same date from a single farm

### Assessment for *T. gondii* antibodies

The serum samples were tested for antibodies against *T. gondii* by modified agglutination test (MAT) [10]. Sera with MAT titers of 1:100 or higher were considered positive for *T. gondii* [1]. Whole formalin-treated *T. gondii* tachyzoite antigens were obtained from the University of Tennessee Research Foundation (Knoxville, TN, USA; https://utrf.tennessee.edu/). *T. gondii*-positive mouse sera were provided by Dr. J. P. Dubey (Beltsville, ARS, USDA) as reference sera. All serum samples were tested at 1:100, then the dilution was doubled to the maximum titer, and positive controls and negative controls were run on each plate.

### Statistical analysis

Statistical analysis was performed using GraphPad Prism 4.0 software (GraphPad Software Inc., San Diego, CA, USA). The data were analyzed using the chi-square test or Fisher’s exact test to assess the association between seropositivity and risk factors based on region (17 cities), season (spring, summer, autumn, and winter), and geographic location (north of the Yellow River, south of the Yellow River).

## Results

Our survey indicated that 1.93% (102/,5292) (95% CI, 1.56–2.30) of the examined dairy cattle were seropositive for *T. gondii* infection by MAT, with titers of 1:100 in 102 cattle, 1:200 in 40, 1:400 in 21, 1:800 in 17, 1:1600 in 12, and 1:3200 in 7 (Table 1). The seroprevalence of *T. gondii* infection in cattle from 17 cities ranged from 0 to 10%. No information on the geographical locations from 756 cattle samples was available. The seroprevalence rates of *T. gondii* varied with regions. The differences in *T. gondii* seroprevalence among different regions are shown in Table 2. A high prevalence was observed in LuoYang and ZhouKou compared to the other regions (*P* < 0.05), and no seropositive sera from cattle were observed in SanMenXia and XinYang.

**Table 2:** *P* values of comparison of seroprevalence of *T. gondii* infection in cattle from different cities by Fisher’s exact test

Location	ZhengZhou	KaiFeng	PingDingShan	LuoYang	AnYang	JiaoZuo	HeBi	XinXiang	PuYang	XuChang	LuoHe	SanMenXia	NanYang	XinYang	ZhouKou	ZhuMaDian
KaiFeng	0.0489^*^	–	–	–	–	–	–	–	–	–	–	–	–	–	–	–
PingDingShan	0.1003	0.4192	–	–	–	–	–	–	–	–	–	–	–	–	–	–
LuoYang	0.0001^*^	0.0790	0.0001^*^	–	–	–	–	–	–	–	–	–	–	–	–	–
AnYang	1.0000	0.0259^*^	0.0398^*^	0.0001^*^	–	–	–	–	–	–	–	–	–	–	–	–
JiaoZuo	0.0867	0.4624	0.7940	0.0001^*^	0.0316^*^	–	–	–	–	–	–	–	–	–	–	–
HeBi	0.0937	0.4263	1.0000	0.0001^*^	0.0271^*^	0.8113	–	–	–	–	–	–	–	–	–	–
XinXiang	0.0484^*^	0.4553	0.8146	0.0001^*^	0.0119^*^	1.0000	0.8350	–	–	–	–	–	–	–	–	–
PuYang	1.0000	0.0851	0.3114	0.0001^*^	0.6271	0.1794	0.1625	0.1476	–	–	–	–	–	–	–	–
XuChang	0.2488	0.3963	1.0000	0.0001^*^	0.1067	0.7744	1.0000	0.7955	0.2856	–	–	–	–	–	–	–
LuoHe	0.3176	1.0000	1.0000	0.1022	0.2541	1.0000	1.0000	1.0000	0.3872	1.0000	–	–	–	–	–	–
SanMenXia	1.0000	0.0473^*^	0.1095	0.0001^*^	1.0000	0.1015	0.1400	0.0751	0.5461	0.1685	0.2500	–	–	–	–	–
NanYang	0.6572	0.1419	0.5244	0.0001^*^	0.3625	0.3433	0.4082	0.2742	1.0000	0.5043	0.4800	0.5545	–	–	–	–
XinYang	1.0000	0.5644	1.0000	0.0938	1.0000	1.0000	1.0000	1.0000	1.0000	1.0000	1.0000	–	1.0000	–	–	–
ZhouKou	0.0073^*^	0.7020	0.0942	0.3615	0.0030^*^	0.1332	0.0855	0.1134	0.0163^*^	0.0845	0.2746	0.0100^*^	0.0322^*^	0.3111	–	–
ZhuMaDian	1.0000	0.3032	0.6889	0.0002^*^	0.5106	0.4450	0.4805	0.4767	1.0000	0.6695	0.4773	0.4662	1.0000	1.0000	0.1256	–
JiYuan	0.0440^*^	0.7051	0.7873	0.0002^*^	0.0139^*^	1.0000	0.8048	0.8105	0.1050	0.5758	1.0000	0.1017	0.2203	1.0000	0.2430	0.4450

*The difference was considered significant when *P* value less than 0.05

Risk factors in relation to geographic location and season were analyzed. The prevalence of *T. gondii* in dairy cattle along the Yellow River was higher than in the other areas (Fig. 1). The seroprevalence of *T. gondii* in cattle from south of the Yellow River (3.67%, 57/2116) was higher than that in the north of the Yellow River (1.69%, 41/2420), with a statistically significant odds ratio of 1.606 (95% CI, 1.071–2.410) (*P* = 0.027). In the summer, no positive sera for *T. gondii* were detected in dairy cattle by MAT (*n* = 416). In spring, *T. gondii* antibodies were identified in 3 (0.71%) of 424 samples. In winter, 14 (1.52%) of 921 serum samples tested positive for *T. gondii* antibodies. Out of the 3531 samples collected in autumn, 85 (2.41%) samples were determined to be positive. Seroprevalence was higher in the autumn in contrast to the spring and winter, and the difference between autumn and spring was statistically significant (OR 3.462; *P* = 0.039) (Table 3).

**Table 3:** Seroprevalence and risk factors of *T. gondii* in dairy cattle in Henan Province

Factor	Category	No. of tested	Number of seropositive samples /(cut-off)						Prevalence (%)	95% Cl
			1:100	1:200	1:400	1:800	1:1600	1:3200		
Geography^a^	North of the Yellow River	2420	41	6	3	3	2	0	1.69%	1.25–2.30
	South of the Yellow River	2116	57	31	18	14	10	7	3.67%	2.08–3.48
Season^b^	Summer	416	0	0	0	0	0	0	–	–
	Spring^a^	424	3	3	2	1	1	1	0.71%	0.14–2.16
	Winter	921	14	2	2	2	2	2	1.52%	0.88–2.56
	Autumn^a^	3531	85	19	17	14	9	6	2.41%	1.95–2.97

^a^*P*-value < 0.05 by two-tailed chi-square tests for geography factor

^b^Spring includes March, April and May. Summer includes June, July, and August. Autumn includes September, October, and November. Winter includes December, January and February

## Discussion

It has been estimated that about one third of the world population has been infected with *T. gondii* [5, 11]. Infection is often most common in areas that have hot, humid climates and lower altitudes [1]. Cattle can be readily infected with *T. gondii*, but they are considered poor hosts because these have developed a more effective immune response to *T. gondii* infection than sheep that possibly facilitates in *T. gondii* elimination from tissues, as well as transient antibody responses [12–14]. However, *T. gondii* has been isolated from cattle tissues or unpasteurized milk [13], indicating that meat and milk may be sources of *T. gondii* infections. *T. gondii* has also been detected in cattle semen [1], suggesting it may be transmitted by venereal contact or artificial insemination.

Several test methods have been used in the diagnosis of *T. gondii* infection in humans. However, these could not be applied to cattle based on its incompatibility with the bovine immunoglobulin G system [15]. Current understanding of the specificity and sensitivity of serological diagnosis of *T. gondii* infection in cattle is limited. Furthermore, cattle may not readily acquire persistent *T. gondii* infections, and the actual prevalence rates based on serum antibodies to *T. gondii* are difficult to ascertain. Previous reports have screened for *T. gondii* antibodies in cattle sera at a 1:100 dilution or higher by MAT [1, 16], prompting us to use this as a cut-off in the present study. Dubey and Jokelainen et al. have conducted *T. gondii* serological testing of cattle [13, 17], and emphasize that the results of *T. gondii* serological screening of cattle should be interpreted with caution. Serology is an indirect method, and the positive results indicate that the host has been exposed to the parasite and produced measurable humoral immune responses. An increase in seroprevalence in cattle up to the age of five years has been reported [17]. The details of the age of the cattle included in the current study were unfortunately unknown, but the age range was wide and did not include only old cattle.

The overall estimated global prevalence of *T. gondii* in cattle using various detection methods is 14.96% (8286/55,377, 1.40–91.80%) [1]. Furthermore, the overall estimated seroprevalence of *T. gondii* in other larger ruminants, specifically water buffalo, horse, and camel was 13.49%, 9.34%, and 35.92%, respectively [1]. The seroprevalence of *T. gondii* based on screening cattle from China is 9.08% (1560/17168, 1.73–46.40%) [18]. In the present study, the seroprevalence of *T. gondii* in cattle was 1.93% (102/5292), which is lower than the rest of the world and China’s average infection rate. It is also lower than the seroprevalence of *T. gondii* in free-range chickens (18.86%, 132/700) [19], ostrich (10.20%, 20/197) [20], sheep (29.33%, 83/283) [21], swine (13.08%, 304/2325) [22], domestic cats (50%, 21/42) [23], and large cats (88.9%, 8/9) [24] in Henan Province. The maximum titer against *T. gondii* antibodies in dairy cattle was 3200 in this study. Considering the effective immune response to *T. gondii* infection in cattle, cattle with high antibody titers may be in the post-acute phase of toxoplasmosis, whereas those with low antibody titers may be in the chronic phase and may contain viable cysts of the parasite in their tissues [1, 25, 26]. However, seronegativity does not guarantee that the meat or milk from cattle will be free of *T. gondii*.

The seroprevalence of 1.93% in this survey indicates that cattle from central China are widely exposed to *T. gondii*. The route of *T. gondii* infection in cattle is probably by ingestion of *T. gondii* oocysts shed by infected felids. This finding suggests that cattle from Henan Province come into contact with *T. gondii* oocysts from cats or from soil, water, or feed. A single *T. gondii* felid could shed millions of oocysts after ingestion of raw meat containing *T. gondii* cysts, and the oocysts can survive in the environment for several years [27]. Furthermore, 100%, 10%, and 71% of cats shed oocysts after primary, secondary, and tertiary infection *T. gondii* [1, 28]. The high seroprevalence of *T. gondii* in domestic cats (50%, 21/42) and wild captured large cats (88.9%, 8/9) from Henan Province [23, 24] suggest that the risk of environmental contamination from felids should be given more attention.

Cattle can acquire *T. gondii* oocysts from the environment by direct contact with soil, feed, or water. The seroprevalence was highest in samples collected in autumn, suggesting that fresh grass used as cattle food may harbor *T. gondii* oocysts, and a humid environment favors the survival of oocysts in this season. The results of the present study agree with those of previous reports [29–31]. However, some studies have shown that season is not a risk factor for *T. gondii* infection [32, 33].

Henan Province is located in the downstream area of the Yellow River. LuoYang and Zhou Kou are much closer to the Yellow River than other cities, and *T. gondii* prevalence in the two regions is relatively higher (*P <* 0.05, Fig. 1). *T. gondii* oocysts can be transported via freshwater runoff into the ocean, thereby posing a threat to humans and other animals residing close to the river [34]. In the present study, risk factor analysis showed that geographic location is associated with *T. gondii* seroprevalence*.* The seroprevalence of *T. gondii* infection in the cattle from south of the Yellow River was higher than that of the north of the Yellow River (*P <* 0.05), which may be attributable to the mountainous geographic environment and higher temperature.

To our knowledge, neither natural cases of toxoplasmosis nor isolated strains of *T. gondii* in Chinese cattle have been reported to date. Cattle are considered poor hosts for *T. gondii* and good hosts for *Neospora caninum*. *N. caninum* infection in cattle has been found to be widespread in central China [35, 36], and this may cause reproductive losses in the cattle [37]. The dominant *T. gondii* genotype in China is ToxoDB#9 [38]. Several studies have identified *T. gondii* ToxoDB#9 in swine, domestic cats, and sheep in Henan Province [21, 23, 24, 39, 40]. However, only ToxoDB#225 *T. gondii* has been identified in Chinese cattle [40]. Additional investigations on cattle toxoplasmosis are thus warranted.

## Conclusions

This is the first large-scale investigation on the seroprevalence of *T. gondii* infection in cattle from central China. The rate of *T. gondii* infection in dairy cattle is relatively low, and this information may be integrated into the Chinese *T. gondii* epidemiology database. Additionally, geographic location appeared as a risk factor for *T. gondii* seropositivity, with prevalence of *T. gondii* in cattle along the Yellow River higher than those of other areas.

## Abbreviations

MAT: Modified agglutination test
PCR: Polymerase chain reaction
